# *Trans*-genetic effects of circular RNA expression quantitative trait loci and potential causal mechanisms in autism

**DOI:** 10.1038/s41380-022-01714-4

**Published:** 2022-08-12

**Authors:** Te-Lun Mai, Chia-Ying Chen, Yu-Chen Chen, Tai-Wei Chiang, Trees-Juen Chuang

**Affiliations:** 1 Genomics Research Center, Academia Sinica, Taipei, 115201 Taiwan; 2 Department of Life Science, National Taiwan University, Taipei, 106319 Taiwan

**Keywords:** Neuroscience, Autism spectrum disorders, Genetics

## Abstract

Genetic risk variants and transcriptional expression changes in autism spectrum disorder (ASD) were widely investigated, but their causal relationship remains largely unknown. Circular RNAs (circRNAs) are abundant in brain and often serve as upstream regulators of mRNAs. By integrating RNA-sequencing with genotype data from autistic brains, we assessed expression quantitative trait loci of circRNAs (circQTLs) that *cis*-regulated expression of nearby circRNAs and *trans*-regulated expression of distant genes (*trans*-eGenes) simultaneously. We thus identified 3619 circQTLs that were also *trans*-eQTLs and constructed 19,804 circQTL-circRNA-*trans*-eGene regulatory axes. We conducted two different types of approaches, mediation and partial correlation tests (MPT), to determine the axes with mediation effects of circQTLs on *trans*-eGene expression through circRNA expression. We showed that the mediation effects of the circQTLs (*trans*-eQTLs) on circRNA expression were positively correlated with the magnitude of circRNA-*trans*-eGene correlation of expression profile. The positive correlation became more significant after adjustment for the circQTLs. Of the 19,804 axes, 8103 passed MPT. Meanwhile, we performed causal inference test (CIT) and identified 2070 circQTL-*trans*-eGene-ASD diagnosis propagation paths. We showed that the CIT-passing genes were significantly enriched for ASD risk genes, genes encoding postsynaptic density proteins, and other ASD-relevant genes, supporting the relevance of the CIT-passing genes to ASD pathophysiology. Integration of MPT- and CIT-passing axes further constructed 352 circQTL-circRNA-*trans*-eGene-ASD diagnosis propagation paths, wherein the circRNA-*trans*-eGene axes may act as causal mediators for the circQTL-ASD diagnosis associations. These analyses were also successfully applied to an independent dataset from schizophrenia brains. Collectively, this study provided the first framework for systematically investigating *trans*-genetic effects of circQTLs and inferring the corresponding causal relations in diseases. The identified circQTL-circRNA-*trans*-eGene regulatory interactions, particularly the internal modules that were previously implicated in the examined disorders, also provided a helpful dataset for further investigating causative biology and cryptic regulatory mechanisms underlying the neuropsychiatric diseases.

## Introduction

Autism spectrum disorder (ASD) is a highly pervasive neurodevelopmental and heritable complex disorder, which is characterized by limited social communication, restricted and repetitive interests or behaviors [1]. Previous large-scale genomic studies have identified a variety of genomic variants associated with ASD etiology [2, 3], providing valuable biological insights into this disorder. However, the contribution of genetic factors to this complex disease is highly heterogeneous and not yet well understood. In addition, through high-throughput RNA sequencing (RNA-seq) data of postmortem brains from people with idiopathic ASD and non-ASD controls, numerous studies identified valuable differential expression patterns in mRNAs [4], long non-coding RNAs [4], microRNAs (miRNAs) [5], and circular RNAs (circRNAs) [6, 7]. While these results increased our understanding of the underlying molecular mechanisms in ASD pathophysiology, the causal relationships between genetic sequence variants and transcriptional expression changes in ASD remain elusive.

By integrating genome-wide genotyping with transcriptome profiling, expression quantitative trait loci (eQTL) analysis identifies single nucleotide polymorphisms (SNPs) that affect the expression levels of local (*cis*-eQTL) or distant (*trans*-eQTL) genes, providing a connection between genetics and regulatory mechanisms of gene expression [8]. *cis*-eQTL SNPs often reside within or close to promoter regions of their nearby genes (*cis*-eGenes) and mediate gene expression through directly affecting the corresponding transcription factor binding process [9]. In contrast to frequent investigation of *cis*-eQTLs, the regulatory mechanisms underlying *trans*-eQTLs are less known because of the relatively weaker effects on gene expression for *trans-*actions than for *cis-*actions and the multiple-testing burden [10]. However, compared with *cis*-effects on gene expression, *trans*-genetic effects were reported to explain more than two times the variability in gene expression [10] and be more cell type-specific [11]. Some disease-causing variants involved in *trans*-effect analyses may miss in *cis*-effect analyses [12]. A recent study further indicated that at least 70% of complex trait heritability is driven by *trans*-eQTL effects [13]. A possible regulatory mechanism for *trans*-eQTLs is that *trans*-eQTLs indirectly affect the expression of distant genes (*trans*-eGenes) through regulating the expression of genes near the *trans*-eQTLs (*cis*-eGenes) [14]. In such cases, a *trans*-eQTL is also a *cis*-eQTLs, wherein the corresponding *cis*-eGene acts as a mediator of the *trans*-eQTL. Investigation of *trans*-genetic effects and the causal inference for the corresponding eQTL-associated networks will allow us to better understand complex trait genetics.

CircRNAs are an emerging class of RNAs, which are formed by pre-mRNA back-splicing with a structure of covalently closed loops and endogenously expressed as single-strand, non-polyadenylated circular molecules [15, 16]. Although circRNAs are generally expressed at a much lower level compared with their corresponding co-linear mRNA isoforms, they are more stable than other types of RNAs [15, 16]. CircRNAs were observed to be especially abundant in mammalian brain tissue and often play important roles in development of nervous system [17]. As for the layer of gene regulatory networks, circRNAs often serve as an upstream regulator of mRNAs. They may regulate genes in *trans* through mediating the activities of miRNAs or RNA binding proteins (RBPs), with the common miRNA/RBP target sites of circRNAs and the target genes [15, 16]. Hence, we are curious about whether SNPs that influence expression of nearby circRNAs (referred to as circQTLs) may also affect expression of remote genes, whereby circRNAs serve as an intermediate regulator bridging circQTLs and *trans*-eGenes. If this is possible, we may generate circQTL-circRNA-*trans*-eGene propagation paths in autistic brains and shed light on the functional consequences of the genetic loci and the etiology of this complex disease.

There are two challenges to achieve the abovementioned goals. First, eQTL analysis requires transcriptomic and genomic data from the same samples with a large sample size. Second, large-scale circRNA study is often hampered by the limitation of the intrinsic circRNA characteristic of lacking polyadenylated tails. The newly released Synapse database [4, 18] comprises a large human brain sample size of both genotype data and RNA-seq data (rRNA-depleted RNAs from total RNAs without poly(A)-selection) from ASD cases and controls, offering an unprecedented opportunity for us to decipher the effects of genetic variation on circRNA expression in ASD brain. We may thereby identify a new reference of circQTLs/*trans*-eQTLs and infer *cis*-mediators of the identified circQTLs (i.e., such circQTLs are also *trans*-eQTLs) and causal effects between circQTLs and ASD diagnosis. Our findings will help to unveil the gap between genetic variation and phenotypic changes in ASD. The designed framework was also applied to the replication analysis of circQTLs (*trans*-QTLs) in schizophrenia (SCZ), supporting the framework for future analyses of eQTLs in neuropsychiatric disease or other complex traits.

## Materials and methods

### Datasets

The rRNA-depleted paired-end RNA-seq data [4] and the corresponding genotype data [18] of postmortem samples from frontal cortex (FC) (Brodmann area 9) and temporal cortex (TC) (Brodmann area 22, 41, and 42) from individuals with ASD and non-ASD controls were obtained from Synapse (http://www.synapse.org) with permission under the accession number syn4587609. The examined circRNAs (1060 circRNAs) were extracted from our previous study [7] based on the same RNA-seq data used in this study. The miRNA expression data based on the 63 cortex samples (30 ASD and 33 non-ASD samples) that overlapped with the samples examined in this analysis were obtained from Wu et al.’s study [5] upon request. For the replication analysis in SCZ, the rRNA-depleted paired-end RNA-seq data (Synapse ID: syn4923029) and the corresponding genotype data (Synapse ID: syn3275221) of postmortem samples from dorsolateral prefrontal cortex from individuals with SCZ and non-SCZ controls were obtained from the CommonMind Consortium (CMC) database [19]. The gene read counts were downloaded from the CMC database (Synapse ID: syn3346749). The examined circRNAs and the back-splicing junction counts were obtained from Liu et al.’s study [20] upon request. The normalization steps of gene/circRNA expression were described in Supplementary Methods.

### Identification of circQTLs/*trans*-eQTLs

We limited our analysis to SNPs in ±200 kb nucleotides of each back-splice site and evaluated circQTLs by testing the correlations between the imputed genotype dosages and covariate-adjusted circRNA expression using Matrix eQTL with an additive linear model [21] (Supplementary Methods). To correct the *P* values regardless of the distribution of circRNA expression and statistical test, a permutation test was conducted to estimate the empirical *P* value (emp*P*) for each uncorrected circQTL SNP by performing 10,000 permutations with randomly sampling labels of the circRNA expression matrix. The emp*P* value for each uncorrected circQTL *P* value (*P*_circQTL_) was calculated as emp*P* = $\frac{{{{{{{{{\mathrm{1 + }}}}}}}}\mathop {\sum }\nolimits_{i = 1}^{{{{{{{{\mathrm{10,000}}}}}}}}} {{{{{{{\mathrm{number}}}}}}}}\;{{{{{{{\mathrm{of}}}}}}}}\;\left( {P_i < P_{circQTL}} \right)}}{{{{{{{{{\mathrm{10,001}}}}}}}}}}.$ To identify circQTL-containing circRNAs, we calculated *q* value for each circRNA using Storey’s method [22] based on the distribution of minimal emp*P* values of all circRNAs. A circRNA was defined as a circQTL-containing circRNA if the *q* value of the circRNA was less than 0.05. For each circQTL-containing circRNA, only the associated circQTLs with emp*P* < 0.005 were retained. We further performed conditional and joint (COJO) analysis based on GCTA-COJO [23] to identify independent circQTLs within highly associated regions.

We examined whether a circQTLs was also a *trans*-eQTL affecting the expression of *trans*-eGenes using Matrix eQTL with an additive linear model. Such circQTL SNPs and *trans*-eGenes should be located on different chromosomes or the same chromosome separated by a distance greater than 5 Mb. To correct the *P* value regardless of the distribution of remote gene expression and statistical test, a permutation test was conducted to estimate the empirical *P* (emp*P*) value for each uncorrected circQTL SNP by performing 10,000 permutations with randomly sampling labels of the expression matrix of remote genes. The *trans*-effect of a circQTL on the expression of a *trans*-eGene was determined if uncorrected *trans*-eQTL *P* value was less than 10^−4^ and emp*P* value was less than 0.005 simultaneously (Supplementary Methods). The *trans*-effects of non-circQTLs on the expression of remote genes were also examined using the same procedures mentioned above.

### Mediation and causal testing

We conducted mediations analysis using the mediation package [24] downloaded from the Comprehensive R Archive Network (CRAN) at https://cran.r-project.org/web/packages/mediation/index.html (Supplementary Methods). For the partial correlation analysis [25], we calculated Spearman correlation coefficient between the normalized expression levels of circRNAs and *trans*-eGenes with controlling for the circQTL (*trans*-eQTL) SNPs. The residuals after adjusting for the circQTLs would be significantly correlated (post-adjustment *P* < 0.05), if there was a causal relationship between circRNA expression and *trans*-eGene expression (i.e., the axes passing the partial correlation test). The CIT package [26] was downloaded from CRAN at https://cran.r-project.org/web/packages/cit/index.html. A CIT-passing circQTL-*trans*-eGene pair (CIT *P* < 0.05 and FDR (*q*) <0.05) represents that the circQTL has a causal effect on the diagnosis status (ASD vs. non-ASD or SCZ vs. non-SCZ) through the *trans*-eGene expression (Supplementary Methods). The FDR (*q*) values were further estimated using CIT-provided permutation test with default parameters.

### Experimental validation

We performed qRT-PCR analyses to examine the correlations between the expression of circHOMER1a, miR-641, and *MBNL3* via in vitro perturbation of circHOMER1a or miR-641 in normal human astrocyte cells (Supplementary Methods). The primer, Dsi-RNA, miRNA mimic, and miRNA inhibitor sequences used in this study were listed in Supplementary Table 1.

## Results

### Identification of circQTLs in ASD brain

**Fig. 1:**
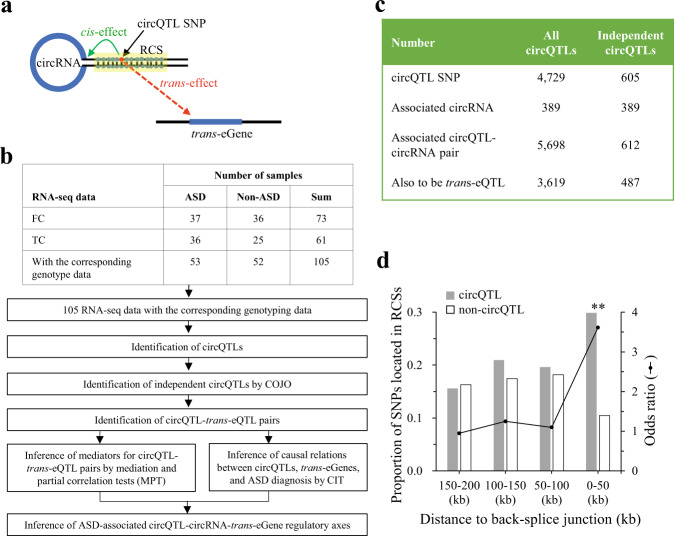
Identification and analysis of circQTLs based on the brain samples from people with ASD and controls. **a** Schematic diagram representing a circQTL that *cis*-regulated expression of a nearby circRNA and *trans*-regulated expression of a distant *trans*-eGene simultaneously. **b** Summary of the workflow for circQTL and causality analyses. **c** The identified circQTLs (all circQTLs and independent circQTLs) and the associated circRNAs according to 105 samples from the cortex (FC and TC). **d** Distance distribution analysis of max-circQTL and non-circQTL SNPs residing in RCSs. *P* values were determined using two-tailed Fisher’s exact test. **P < 0.01. FC frontal cortex. TC temporal cortex.

The goal of this study is to investigate circQTLs that were also *trans*-eQTLs affecting the expression of distant genes (*trans*-eGenes) (Fig. 1a). We collected RNA-seq data [4] and the corresponding genotype data [18] of postmortem samples from the cortex (FC/TC) from individuals with ASD and non-ASD controls (Fig. 1b; Supplementary Data 1). The examined circRNAs (Supplementary Data 1) were extracted from our previous study [7] based on the same RNA-seq data used in this study. CircQTLs were evaluated by testing the correlations between the imputed genotype dosages and covariate-adjusted circRNA expression using Matrix eQTL [21]. After permutation and *q* value correction, we identified 4729 circQTLs associated with 389 circRNAs and constructed 5698 circQTL-circRNA associations (Fig. 1c). To determine independent circQTLs within highly associated regions, the COJO analysis [23] identified 605 independent circQTLs associated with 389 circRNAs (Fig. 1c; Supplementary Data 2).

We then studied the relationship between circQTLs and circRNA formation. We found that the majority of the independent circQTLs (578 out of 605; Supplementary Data 2) were located in the flanking sequences of back-splice sites. It was shown that back-splicing can be facilitated by reverse complementary sequences (RCSs) residing in the introns flanking circularized exons [27, 28]. Regarding the most significant circQTL SNP (max-circQTL) for each circRNA, distance distribution analysis revealed that the max-circQTLs residing in RCSs were preferentially located in the flanking sequences close to the back-splice donor/acceptor sites by using the minor allele frequency- and distance-matched non-circQTL SNPs as controls (Fig. 1d). Of note, the examined non-circQTL SNPs were also located in the flanking sequences of the examined back-splice sites and not determined as circQTLs with minimal uncorrected *P* values larger than 0.05 (Supplementary Methods). This result suggests that circQTLs located in the flanking sequences of back-splice sites may contribute to circRNA formation.

### Mediation effects of *trans*-eQTLs (circQTLs) via circRNA expression

**Fig. 2:**
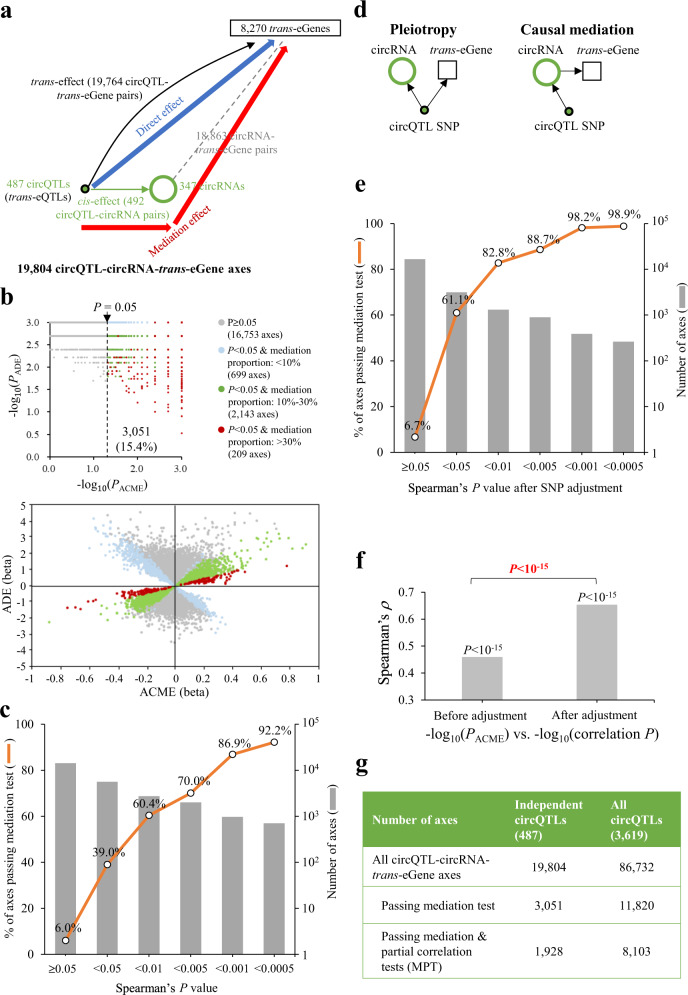
Mediation analysis for the identified circQTL-circRNA-*trans*-eGene axes based on the independent circQTLs. **a** Schematic diagram representing direct (blue bold line with arrow) and mediation (red bold line with arrow) effects of circQTLs (*trans*-eQTLs) on the expression of *trans*-eGenes. **b** The significance levels (top) and effect sizes (beta values; bottom) of the direct (y axis) and mediation (x axis) effects for the 19,804 circQTL-circRNA-*trans*-eGene axes. *P*_ADE_ and *P*_ACME_ values represented the significance levels of the average direct (ADE) and average causal mediation (ACME) effects, which were determined by the mediation package [24]. The dashed line represented *P* = 0.05 for mediation effects. Of the 19,804 circQTL-circRNA-*trans*-eGene axes, 3051 axes (15.4%) passed the mediation test. For the top right panel, the numbers of axes were shown in parentheses. The correlations between the percentages of circQTL-circRNA-*trans*-eGene axes passing the mediation test and the significance levels of the Spearman’s correlation of expression profile between circRNAs and *trans*-eGenes before (**c**) and after (**e**) SNP adjustment. **d** Two possible explanations for the association between circRNA expression and *trans*-eGene expression through genetic variants. **f** Correlations between mediation effects and the significance levels of circRNAs-*trans*-eGenes correlations before or after SNP adjustment. Statistical significance of Spearman’s rank correlation coefficient was denoted by black words. Significant difference between two independent correlations was evaluated using two-tailed Z-score test and denoted by red words. **g** A summary table of the identified circQTL-circRNA-*trans*-eGene axes passing mediation test or MPT.

We next examined whether the identified circQTLs were also *trans*-eQTLs affecting the expression of *trans*-eGenes. Of the 605 independent circQTLs, 487 circQTLs (associated with 347 circRNAs) were identified to be also *trans*-eQTLs affecting the expression of 8270 *trans*-eGenes (Fig. 2a; Supplementary Data 3). We thus constructed 19,804 circQTL-circRNA-*trans*-eGene axes. These circQTL-circRNA-*trans*-eGene axes also resulted in 18,863 circRNA-*trans*-eGene pairs, wherein the expression levels of circRNAs and *trans*-eGenes were both regulated through the same circQTLs (Fig. 2a). We also examined the *trans*-effect of non-circQTLs on the expression of distant genes. We found that max-circQTLs had a significantly higher percentage of *trans*-eQTLs than non-circQTLs (81.7% vs. 70.4%; *P* < 0.001 by two-tailed Fisher’s exact test), suggesting that circQTLs were more likely to be also *trans*-eQTLs as compared with non-circQTLs.

We then explored the causes for the *trans*-effect of circQTLs on the expression of *trans*-eGenes. In addition to the direct effect of circQTLs on *trans*-eGene expression, we hypothesized that in some cases the expression of *trans*-eGenes were indirectly regulated through the expression of circRNAs near the circQTLs (“mediation effect”; Fig. 2a). For the scenario of mediation effects, the circRNAs served as *cis*-mediators of *trans*-eQTLs. To test the mediation effects of *trans*-eQTLs, we performed mediation analyses [24] for the 19,804 circQTL-circRNA-*trans*-eGene axes to identify the proportion of association between a circQTL and a *trans*-eGene that was caused by the effect of the circQTL on the corresponding circRNA expression. We found that 3051 out of the 19,804 (15.4%) axes passed the mediation test (Supplementary Data 3), which were significantly mediated by the expression of circRNAs near the circQTLs. The majority (77.1%, 2352 axes) of the 3051 axes exhibited that at least 10% of the circQTL-*trans*-eGene associations were mediated by the expression of circRNAs; in some cases (209 axes), the proportion of mediation of the circQTL-*trans*-eGene association by the circRNAs was more than 30% (Fig. 2b).

We next examined the relationship between mediation effects and the significance levels of circRNA-*trans*-eGene correlation of expression profile. We found that 39% of the circQTL-circRNA-*trans*-eGene axes passed the mediation test if the circRNA expression and *trans*-eGene expression were significantly correlated with each other (Spearman’s *P* < 0.05), whereas such a percentage was significantly reduced from 39% to 6% if the circRNA expression and *trans*-eGene expression were uncorrelated (Fig. 2c). The percentages of the circQTL-circRNA-*trans*-eGene axes passing the mediation test markedly increased with increasing significance levels of the correlation of expression profile between circRNAs and *trans*-eGenes (Fig. 2c). Such a percentage even reached 92% at Spearman’s *P* < 0.0005. This result indicated that the mediation effects of the circQTLs (*trans*-eQTLs) on circRNA expression were positively correlated with the magnitude of circRNA-*trans*-eGene correlation of expression profile, implying a causal relationship between circRNA expression and *trans*-eGene expression. We thus speculated that there was a residual correlation between circRNA expression and *trans*-eGene expression after repressing out the variance attributable to the circQTL SNP. We conducted a partial correlation analysis by performing Spearman correlation coefficient between circRNA expression and *trans*-eGene expression with adjustment for the circQTLs. The residuals after removing the variance due to the circQTLs would be uncorrelated, if the SNPs had independent associations with circRNA expression and *trans*-eGene expression (the hypothesis of pleiotropy; Fig. 2d, left). In contrast, the partial correlation would be statistically significant after SNP adjustment, if there was a causal relationship between circRNA expression and *trans*-eGene expression (the axes passing the partial correlation test; Fig. 2d, right). We found that 61% of the circQTL-circRNA-*trans*-eGene axes passed the mediation test if the circRNA-*trans*-eGene pairs showed significant correlations after adjustment for the circQTLs (post-adjustment *P* < 0.05; Fig. 2e). This percentage was significantly higher than that derived from the correlation analysis before SNP adjustment (61% vs. 39%; Fig. 2c, e). Such a percentage even reached 99% at post-adjustment *P* < 0.0005. The *P* values of average causal mediation effects (*P*_ACME_) estimated by the mediation test were more strongly correlated with the post-adjustment *P* values than with the correlation *P* values before SNP adjustment (Fig. 2f). These results revealed that some circQTL-circRNA-*trans*-eGene axes, particularly the axes passing both the mediation and partial correlation tests (designated “MPT”), were in the cases wherein the circQTL-*trans*-eGene associations were mediated by the expression of circRNAs. Considering all circQTLs (4729 circQTLs, Fig. 1c), 3619 circQTLs were also to be *trans*-eQTLs and associated with 86,732 circQTL-circRNA-*trans*-eGene axes; of the 86,732 axes, 11,820 passed the mediation test and 8103 passed MPT (Fig. 2g; Supplementary Data 3).

Replication analysis was conducted in an independent dataset from individuals with SCZ and non-SCZ controls (Supplementary Fig. 1; Supplementary Data 4). Like the trends observed in the analyses for the ASD samples, the mediation effects of the circQTLs (*trans*-eQTLs) on circRNA expression were positively correlated with the magnitude of circRNA-*trans*-eGene correlation of expression profile, regardless of whether the circQTL SNPs were controlled (Supplementary Methods and Supplementary Fig. 2). The *P*_ACME_ values were more strongly correlated with the post-adjustment *P* values than with the correlation *P* values before SNP adjustment, also supporting the mediation effects of *trans*-eQTLs for the SCZ samples.

### The associations of the identified circQTL-circRNA pairs with ASD

The recent large-scale genome-wide association studies (GWASs) have identified numerous high-confidence genetic risk loci for ASD [2]. To detect the associations of the identified circQTL-circRNA pairs with ASD, we assessed the overlap between the internal modules of the pairs (circQTLs/circRNAs) and ASD-related GWAS sites/SNP clumps (Supplementary Methods; Supplementary Fig. 3a). Of the identified circQTLs, 342 were ASD-related GWAS SNPs (*P* < 0.05). Although none of the circQTLs was significantly ASD-related GWAS sites (*P* < 5 × 10^−8^), 168 circQTLs were located within the ASD-associated SNP clumps. Regarding the circRNAs of the identified circQTL-circRNA pairs, the back-splice sites (donor or acceptor sites) of 106 circRNAs overlapped with the ASD-associated SNP clumps (Supplementary Fig. 3a). In addition, 52 circRNAs were previously identified to be differentially expressed in ASD brain (“DE-circRNAs” [7]) or involved in a circRNA coexpression module upregulated in ASD samples (“DE-circRNA module” [6]). Of note, both the DE-circRNAs and DE-circRNA module were identified with the same cortex samples examined in this study. For the replication analysis of the SCZ samples, we extracted the GWAS data for SCZ [29]. We found that 74,029 circQTLs were SCZ-related GWAS SNPs (2892 were significantly SCZ-related GWAS sites) and 90,746 circQTLs were located within SCZ-associated SNP clumps (Supplementary Fig. 3b; Supplementary Data 4). For the corresponding circRNAs, the back-splice sites of 4802 circRNAs overlapped with the SCZ-associated SNP clumps; and 134 circRNAs were identified to be DE-circRNAs in SCZ brain (Supplementary Methods; Supplementary Data 6). Moreover, we performed a formal transcriptome-wide association study (TWAS) to determine circRNAs whose *cis*-regulated expression was associated with diseases using TWAS-FUSION [30] (“FUSION”). While no circRNAs were prioritized by FUSION in the ASD samples, 35 circRNAs were identified to be TWAS-significant circRNAs in the SCZ samples (Bonferroni-corrected *P* < 0.05; Supplementary Fig. 3c; Supplementary Data 7). Taken together, these results provided a core set of candidate circQTLs and circRNAs implicated by risk loci. The associations of circQTLs and circRNAs with the examined diseases were summarized in Supplementary Fig. 4.

### Causal effects between circQTLs (*trans*-eQTLs) and ASD

**Fig. 3:**
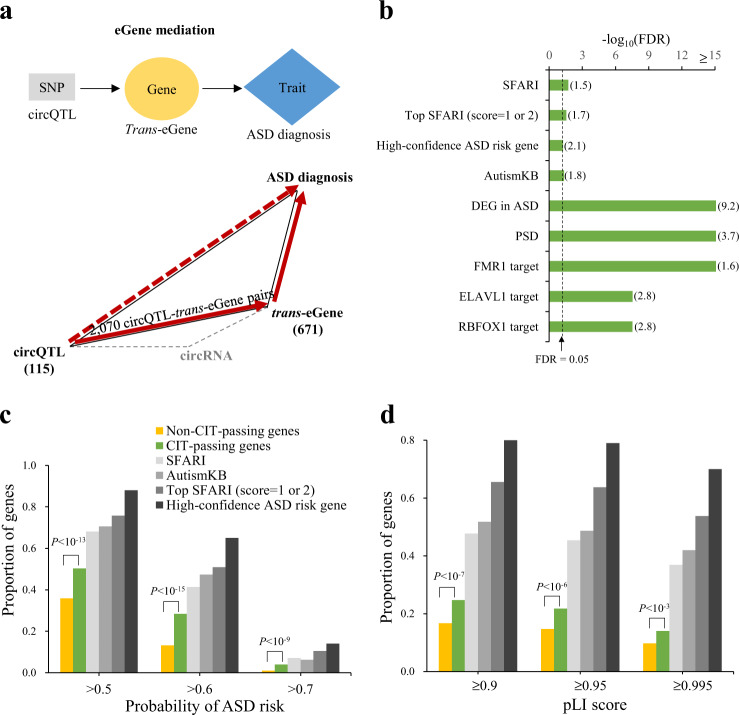
Inference of causal relations between circQTLs (*trans*-eQTLs), *trans*-eGenes, and ASD diagnosis by CIT. **a** The circQTL-*trans*-eGene pairs (2070 pairs) passing CIT with the propagation path from circQTL SNPs (115 circQTLs) to ASD diagnosis via *trans*-eGene (671 genes) expression. **b** Enrichment analysis of ASD-relevant genes for the CIT-passing genes. ASD-relevant genes included ASD risk genes (from SFARI, AutismKB, and high-confidence ASD genetic risk genes identified by Satterstrom et al. [3]), DEGs in ASD, genes encoding postsynaptic density (PSD) proteins, genes encoding transcripts bound by FMR1 (FMR1 target), RBFOX1 (RBFOX1 target), and ELAVL1 (ELAVL1 target). Top SFARI genes represented SFARI genes with score = 1 or 2. Comparisons of the proportions of CIT-passing genes and non-CIT-passing genes with different probabilities of ASD risk **c** and different gene variant intolerance (pLI) scores **d**. All *P* values were determined using one-tailed Fisher’s exact test. For **b**, *P* values were FDR adjusted across nine target groups for each gene list using Benjamini-Hochberg correction. The dashed lines represented FDR *=* 0.05. The enrichment odd ratios with FDR ≤ 0.05 were shown in parentheses.

We then conducted a causal inference test [26] (CIT; Materials and Methods) to examine whether the *trans*-effects of circQTLs on *trans*-eGene expression might explain the association between the circQTLs and diagnosis status (ASD vs. non-ASD) and infer the direction of association between circQTLs, *trans*-eGene expression, and ASD diagnosis (Fig. 3a). Regarding all the identified circQTLs that were also *trans*-eQTLs and the associated circQTL-*trans*-eGene pairs in the ASD samples, we identified 2070 circQTL-*trans*-eGene pairs with a propagation path from circQTL SNPs to ASD diagnosis via *trans*-eGene expression (Fig. 3a; Supplementary Data 3). We speculated that the *trans*-eGenes (671 genes; CIT-passing genes) involved in the 2070 circQTL-*trans*-eGene pairs may be implicated in ASD. We examined enrichment analysis for the genes previously implicated in ASD from Simons Foundation Autism Research Institutive (SFARI) [31], the high-confidence ASD genetic risk genes [3], and Autism KnowledgeBase (AutismKB) [32]. Indeed, the CIT-passing genes were significantly enriched for these ASD risk genes (Fig. 3b). These CIT-passing genes were also enriched for other classes of ASD-relevant genes, including genes encoding postsynaptic density (PSD) proteins [33], genes whose transcripts were bound by the RBPs (FMR1 [34], RBFOX1 [35], and ELAVL1 [36]), and differentially expressed genes (DEGs) in ASD [4] that were derived from the same cortex samples used in this study (Fig. 3b). Gene Ontology (GO) analysis revealed that the CIT-passing genes were enriched in GO terms related to metal ion transmembrane transporter activity, chemical synaptic transmission, synaptic signaling, neuron development, neurogenesis, synapse, neuron projection, and axon (Supplementary Fig. 5), further reflecting their enrichment for PSD. Moreover, a previous study has presented a genome-wide prediction of ASD risk genes and provided an estimated probability of ASD-association for each gene [37]. The CIT-passing genes indeed had a significantly higher probability of ASD risk compared with non-CIT-passing genes (Fig. 3c). Furthermore, the CIT-passing genes were more intolerant of a loss function mutation (measured by gene variant intolerance (pLI) scores [38]) compared with non-CIT-passing genes (Fig. 3d), reflecting previous observations that genes implicated in ASD tended to be subject to stronger selective constraints than the other genes [37]. These observations support the relevance of the CIT-passing genes to ASD pathophysiology. Similarly, applying the CIT analysis to the identified circQTL-*trans*-eGene pairs in the SCZ samples (Supplementary Data 5) showed that the CIT-passing genes were significantly enriched for DEGs in SCZ and the genes previously implicated in SCZ (Supplementary Fig. 6).

### Identification of circQTL-circRNA-*trans*-eGene-disease propagation paths

**Fig. 4:**
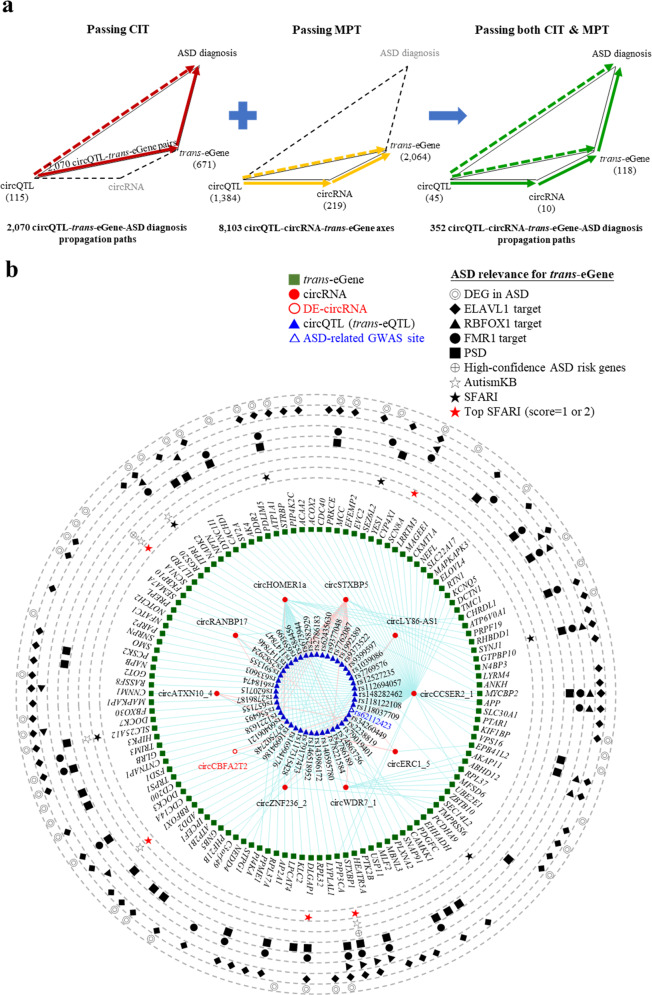
Potential circQTL-circRNA-*trans*-eGene-ASD diagnosis propagation paths. **a** Identification of potential circQTL-circRNA-*trans*-eGene-ASD diagnosis propagation paths by integrating CIT-passing circQTL-*trans*-eGene pairs with MPT-passing circQTL-circRNA-*trans*-eGene axes. **b** The 352 identified circQTL-circRNA-*trans*-eGene-ASD diagnosis propagation networks plotted by the Cytoscape package. The ASD-relevant circQTL SNP, circRNA, and *trans*-eGenes were shown.

The above mediation and partial correlation analyses (MPT) had identified 8103 circQTL-circRNA-*trans*-eGene axes with significant mediation effects of circQTLs on *trans*-eGene expression through circRNA expression (Fig. 2g). By integrating the 2070 CIT-passing circQTL-*trans*-eGene pairs (Fig. 4a, left) with the 8103 circQTL-circRNA-*trans*-eGene axes (Fig. 4a, middle), we determined 352 circQTL-circRNA-*trans*-eGene-ASD diagnosis propagation paths, wherein the circRNA-*trans*-eGene axes may act as causal mediators for the circQTL-ASD diagnosis associations (Fig. 4a, right; Supplementary Data 3). The 352 ASD-associated circQTL-circRNA-*trans*-eGene axes involved 45 circQTLs (*trans*-eQTLs), 10 circRNAs, and 118 *trans*-eGenes (Fig. 4a, b), of which one circQTL was an ASD-related GWAS SNP, one circRNA was a DE-circRNA in ASD, and 101 of 118 (86%) *trans*-eGenes were ASD-relevant genes. Of the 352 circQTL-circRNA-*trans*-eGene axes, 278 (79%) were associated with the 101 ASD-relevant genes. Particularly, of the 101 ASD-relevant genes, 47 were DEGs in ASD and 11 were SFARI genes (five were top SFARI genes), suggesting the regulatory role of the corresponding circQTL-circRNA-*trans*-eGene axes in ASD brain. Similarly, in SCZ, 263 circQTL-circRNA-*trans*-eGene-SCZ diagnosis propagation paths that passed both CIT and MPT were conducted (Supplementary Fig. 6; Supplementary Data 5).

### Potential mediators for the circRNA-*trans*-eGene interactions

**Fig. 5:**
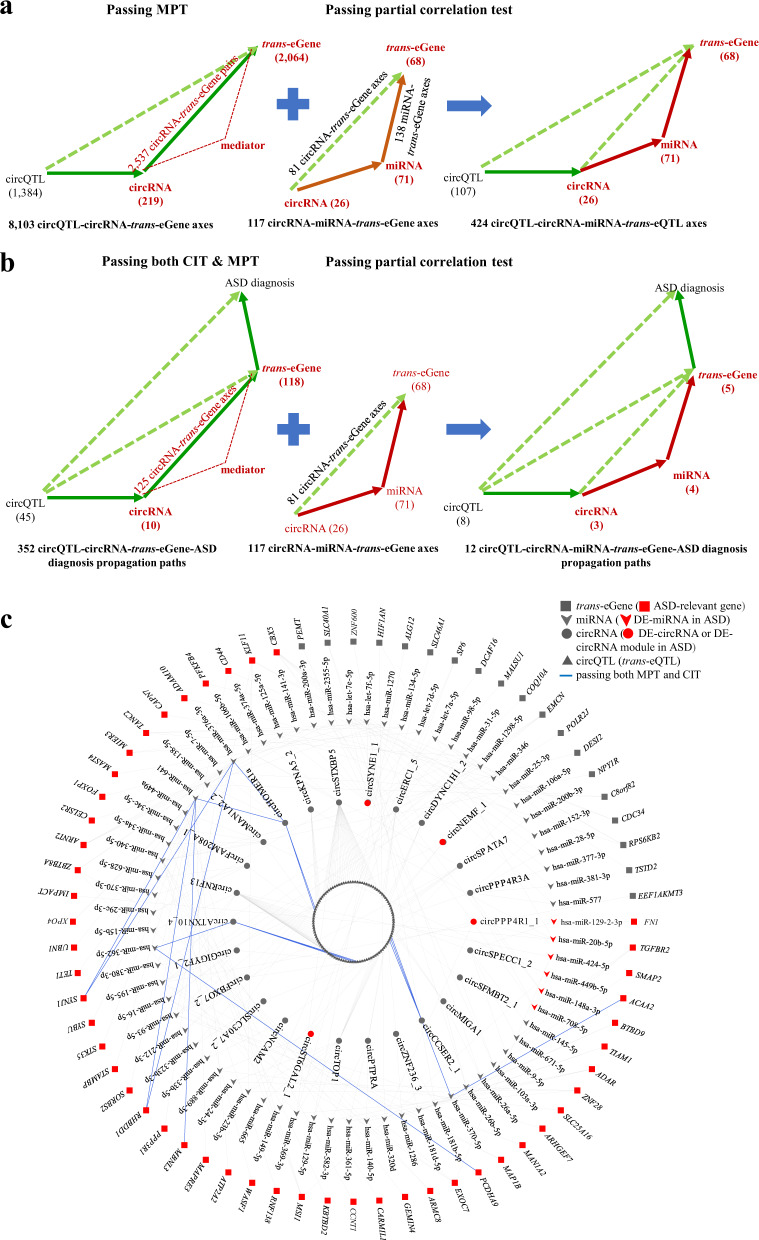
Potential mediators for the circRNA-*trans*-eGene interactions. **a** Identification of potential circQTL-circRNA-miRNA-*trans*-eGene propagation paths by integrating the 8103 MPT-passing circQTL-circRNA-*trans*-eGene axes (see also Fig. 5a) with the 117 circRNA-miRNA-*trans*-eGene axes passing the conducted partial correlation test (see text for details). **b** Identification of potential circQTL-circRNA-miRNA-*trans*-eGene-ASD diagnosis propagation paths by integrating the 352 circQTL-circRNA-*trans*-eGene-ASD diagnosis propagation paths (see also Fig. 5) with the 117 circRNA-miRNA-*trans*-eGene axes illustrated in (**a**). **c** The 424 identified circQTL-circRNA-miRNA-*trans*-eGene propagation networks (see (**a**)) plotted by the Cytoscape package. The 12 circQTL-circRNA-miRNA-*trans*-eGene-ASD diagnosis propagation paths (see (**b**)) were highlighted with blue bold lines.

Regarding the 8103 MPT-passing circQTL-circRNA-*trans*-eGene axes (associated with 2537 circRNA-*trans*-eGene pairs; Fig. 5a, left), we proceeded to identify potential mediators for the circRNA-*trans*-eGene interactions. Numerous cases of circRNAs were demonstrated to act as an upstream regulator of mRNAs through mediating miRNA activities [15, 16]. We thus performed the following steps to determine the circRNA-miRNA-*trans*-eGene axes, wherein the miRNAs may act as a mediator for the circRNA-*trans*-eGene associations. First, we utilized crosslinking immunoprecipitation (CLIP)-seq data-supported RNA interactomes (extracted from ENCORI [39]) to search for the common miRNA target sites of the 2537 circRNA-*trans*-eGene pairs (Supplementary Methods). Second, we extracted the miRNA expression data [5] from the cortex samples that overlapped with the samples examined in this analysis. Only the circRNA-miRNA-*trans*-eGene axes were retained, if the circRNA expression was significantly correlated with the *trans*-eGene expression (*P* < 0.05 by Spearman’s correlation test). Third, we reevaluated the above correlations by performing a partial correlation analysis to control for the miRNA expression. If the miRNAs acted as a mediator for the circRNA-*trans*-eGene associations, the residuals after adjusting for the miRNA expression would be unrelated. We thus identified 117 circRNA-miRNA-*trans*-eGene axes passing the abovementioned partial correlation test, which were associated with 26 circRNAs (4 were DE-circRNAs or involved in the DE-circRNA module), 71 miRNAs (6 DE-miRNAs), and 68 *trans*-eGenes (49 ASD-relevant genes) (Fig. 5a, middle; Supplementary Data 8). Of the 117 circRNA-miRNA-*trans*-eGene axes, 34 axes had the miRNA-*trans*-eGene interactions supported by other experimental data collected in miRTarBase [40] or DIANA-TarBase [41] (Supplementary Data 8). By integrating the MPT-passing circQTL-circRNA-*trans*-eGene axes with the 117 circRNA-miRNA-*trans*-eGene axes, we further conducted 424 circQTL-circRNA-miRNA-*trans*-eQTL axes (Fig. 5a, right). In these propagation paths, the circRNA-miRNA axes may play a causal mediator for the circQTL-*trans*-eGene associations. Particularly, 9 *trans*-eGenes of the propagation paths were previously reported to be ASD risk genes (SFARI or AutismKB genes; Supplementary Data 8). It would be possible to further conduct circQTL-circRNA-miRNA-*trans*-eQTL axes that might be causally related to ASD diagnosis by integrating the 117 circRNA-miRNA-mRNA axes with the 352 abovementioned circQTL-circRNA-*trans*-eGene-ASD diagnosis propagation paths that passed both MPT and CIT (Fig. 5b). Finally, 12 circQTL-circRNA-miRNA-*trans*-eGene-ASD diagnosis propagation paths were identified (Fig. 5b, c; Supplementary Data 8), further providing potentially causal relationships between genetics and trait and the corresponding regulatory mechanisms underlying ASD.

**Fig. 6:**
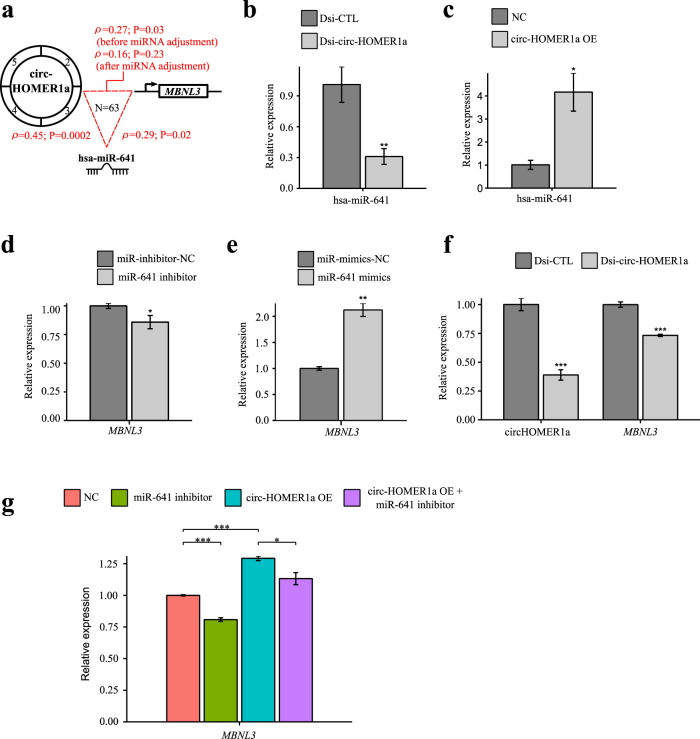
Experimental validation of the correlations between the expression of circHOMER1a, miR-641, and *MBNL3*. **a** Spearman’s correlations between the expression of circHOMER1a, miR-641, and *MBNL3* on the basis of the 63 cortex samples (30 ASD and 33 non-ASD samples). The partial correlation analysis by performing Spearman correlation coefficient between circRNA expression and *trans*-eGene expression with adjustment for the miRNA expression was also shown. The circularized exons of circHOMER1a included Exons 2-5. qRT-PCR analysis of the expression of circHOMER1a and miR-641 after circHOMER1a knockdown **b** or overexpression **c** in normal human astrocyte (NHA) cells. The expression levels of miR-641 were normalized to *U6*. qRT-PCR analysis of the expression of miR-641 and *MBNL3* after miR-641 knockdown **d** or mimics **e** in NHA cells. **f** qRT-PCR analysis of *MBNL3* expression after circHOMER1a knockdown in NHA cells. **g** qRT-PCR analysis of *MBNL3* expression after miR-641 inhibition, circHOMER1a overexpression, and circHOMER1a overexpression with miR-641 inhibition, respectively. **d**–**g** The expression levels of *MBNL3* were normalized to *GAPDH*. **b**–**f** Data were shown as the means±standard deviation of three experiments. *P* values were determined using two-tailed *t*-test assuming unequal variance. *P < 0.05, **P < 0.01, ***P < 0.001. OE overexpression. NC negative control.

Intriguingly, the 12 ASD-associated propagation paths involved a psychiatric disease-associated circRNA (circHOMER1a), which was previously demonstrated to play an important regulatory role in synaptic gene expression and cognitive flexibility [42]. Spearman’s correlation analysis showed that the expression levels of circHOMER1a, miR-641, and *MBNL3* were significantly positively correlated with each other (Fig. 6a; Supplementary Data 8). We examined the correlations via in vitro perturbation of circHOMER1a or miR-641 in normal human astrocyte cells (Supplementary Methods) and validated that the expression levels of circHOMER1a and miR-641 were positively correlated with that of *MBNL3* (Fig. 6b–f). The circHOMER1a-mediated enhancement of *MBNL3* was significantly attenuated by the knockdown of miR-641 (Fig. 6g), reflecting our result of partial correlation test.

## Discussion

This study focused on detecting the circQTLs that *cis*-regulated expression of nearby circRNAs and *trans*-regulated expression of distant genes simultaneously. We asked whether in some cases the effects of *trans*-eQTLs on *trans*-eGene expression were mediated by the circRNA expression. We first performed the mediation analysis (a linear model) and suggested that a considerable percentage of the identified circQTL-*trans*-eGene associations were mediated by the corresponding circRNA expression. We found that the mediation effects of the circQTLs (*trans*-eQTLs) on circRNA expression significantly increased with increasing significance levels of the correlation of expression profile between circRNAs and *trans*-eGenes (Fig. 2c). In addition to the mediation test, we also conducted a partial correlation analysis based on a nonparametric statistic (i.e., residual correlation after regressing out the variance attributable to the circQTL SNPs) to examine the scenario of a causal relationship between circRNA expression and *trans*-eGene expression. The percentages of the circQTL-circRNA-*trans*-eGene axes passing the mediation test increased with increasing the post-adjustment *P* values (Fig. 2e), revealing a high degree of overlap between the results of the mediation test and the partial correlation test. These observations support the possible regulatory mechanism for the circQTLs, wherein circQTLs *cis*-regulate the expression of their nearby circRNAs and consequently regulate the *trans*-eGene expression through mediating the circRNA expression. However, it remains challenging to assess definitive evidence of causality for mediator-outcome pairs because hidden mediator-outcome confounding factors may introduce uncertainty into the results of causality analyses [43]. The discrepancies in the results between these two different types of approaches (Supplementary Fig. 7) reflect the probability of bias in the causality analyses. Therefore, we suggest that the circQTL-circRNA-*trans*-eGene axes passing both tests should be more likely to be in the case of mediation effect compared with the axes passing a single test only.

Generally, there are three possible models (eGene mediation model, reverse causality model, and independent model) to explain the relationships between an eQTL, an eGene, and a trait [14, 30]. For the first model, the eGene acts as a causal mediator for the eQTL-trait associations, wherein the eQTL regulates the trait through mediating the eGene expression. For the second model, the trait acts as a causal mediator, wherein alteration of the eGene expression is the consequence of the trait. For the third model, the effects of eQTLs on eGene expression and trait are independent. In this study, with genomic, transcriptomic, and trait (e.g., ASD vs. non-ASD) data from the same samples, it was possible to assess eGenes that were causally related to idiopathic ASD (i.e., the first model). We utilized CIT to identify 2070 circQTL-*trans*-eGene pairs with *trans*-eGenes as a potentially causal mediator for ASD diagnosis (Fig. 3a). According to the criteria of CIT, a CIT-passing pair should simultaneously satisfy four conditions (Supplementary Methods), especially for the two conditions: circQTLs should be independent of ASD diagnosis after adjusting for *trans*-eGene expression and *trans*-eGene expression should be correlated with ASD diagnosis after adjusting for circQTLs. Therefore, upon passing the CIT screening, both reverse causality and independent models are less probable for the explanation of the CIT-passing circQTL-*trans*-eGene associations.

On the basis of the GWAS data for ASD or SCZ, we found that the numbers of circQTLs and circRNAs overlapping with disease-related GWAS SNPs/clumps were remarkably larger in the SCZ samples than in the ASD samples (Supplementary Fig. 3). The reasons may be due to two inherent limitations of the GWAS data. First, the number of SCZ-related SNPs in the SCZ GWAS was remarkably larger than that of ASD-related SNPs in the ASD GWAS. Particularly, while 22,344 SNPs were identified to be significantly associated with SCZ (GWAS *P* < 5 × 10^−8^) in the SCZ GWAS, only 93 significantly ASD-related SNPs were detected in the ASD GWAS (Supplementary Fig. 3). Second, the significance levels of disease-related SNPs were generally higher in the SCZ GWAS than in the ASD GWAS (Supplementary Fig. 3). This also reflected that dozens of TWAS-significant circRNAs were prioritized in the SCZ samples but no TWAS-significant circRNA was identified in the ASD samples.

Regarding the circQTLs in the identified circQTL-circRNA-*trans*-eGene axes, we observed that 291 circQTLs overlapped with ASD-related GWAS SNPs (Supplementary Fig. 4b and Supplementary Data 3). If the ASD-related circQTLs have high *trans*-effects on the expression of the corresponding *trans*-eGenes, we would speculate that the *trans*-eGenes may play a role in ASD development. Indeed, we observed some circQTLs with high effects (e.g., beta values>1 or <-1) on *trans*-eGene expression, in which the corresponding *trans*-eGenes included 21 SFARI genes and 14 DEGs in ASD (Supplementary Fig. 8 and Supplementary Data 3). It is worthwhile to further investigate the relevance of the corresponding *trans*-eGenes to ASD pathophysiology. On the other hand, the identified circQTL-circRNA-*trans*-eGene axes involved 732 *trans*-eGenes (Supplementary Fig. 4b and Supplementary Data 3) that were ASD-associated DEGs [4] derived from the same cortex samples used in this study. A circQTL that can significantly explain the expression variation of the DEGs in ASD implies the causal relationship between the circQTL and ASD. Considering the maximum variance (*r*^*2*^) explained by a circQTL for each DEG, we observed that the *r*^*2*^ values ranged from 0.14 to 0.38 (Supplementary Fig. 9 and Supplementary Data 3). This result reveals that these ASD-associated genes can be to a certain extent explained by the circQTLs, suggesting the causality of the circQTLs and autism.

Using the miRNA expression data from the same samples examined in this analysis, we identified 12 circQTL-circRNA-miRNA-*trans*-eGene-ASD diagnosis propagation paths (Fig. 5b), wherein the circRNA-*trans*-eGene axes may act as causal mediators for the circQTL-ASD diagnosis associations (passing CIT and MPT) and the miRNAs may act as mediators for the circRNA-*trans*-eGene associations (passing the partial correlation test). One of the 12 axes (circHOMER1a-miR-641-*MBNL3*) involved a psychiatric disease-associated circRNA (circHOMER1a), which was previously reported to be important in regulating synaptic gene expression and cognitive flexibility [42]. Spearman’s correlation analysis (Fig. 6a) and the subsequent validations (Fig. 6b–g) showed positive correlations between the expression levels of circHOMER1a, miR-641, and *MBNL3* and the mediation effect of miR-641 on the circHOMER1a-miR-641-*MBNL3* association. Such a correlation pattern is similar to the previously validated interactions between circCSNK1G3, miR-181b/d, and *CDK1*/*CDC25A* in PC-3 cells [44]. While the best understood regulatory role of circRNAs in miRNA activities is miRNA sponges [15, 16], these results support the miRNA stabilization mechanism for circRNAs [16, 44, 45]. Of note, *MBNL3* is known as a splicing factor, which can regulate numerous alternative polyadenylation events [46]; meanwhile, dysregulation of alternative polyadenylation was suggested to contribute to the pathology of ASD [47]. The functional interaction of this propagation path and the corresponding mechanism underlying ASD are worthy of further investigations.

According to a large brain sample size of both RNA-seq data and the corresponding genotype data from ASD cases and controls, this study is the first report, to the best of our knowledge, for systematically investigating *trans*-genetic effects of circQTLs and inferring the corresponding causal relations in diseases. Our replication analysis in SCZ samples also supports the potential of the provided framework for future investigation of eQTL-trait relations in other complex diseases. Integration of the relevance of the individual internal modules (circQTLs, circRNAs, and *trans*-eGenes) of the identified axes to ASD (or SCZ) provides a helpful dataset for further investigating the cryptic regulatory mechanisms underlying these neuropsychiatric diseases (Supplementary Fig. 4). Future studies with larger patient cohorts, including genome-wide transcriptomic and genomic sequencing data, will allow for more precise causal relationships between genetic variants and transcriptional expression changes in the examined disorders.

## Supplementary information

Supplementary Information

Supplementary Data 1

Supplementary Data 2

Supplementary Data 3

Supplementary Data 4

Supplementary Data 5

Supplementary Data 6

Supplementary Data 7

Supplementary Data 8

## Data Availability

The RNA-seq and genotype data of ASD and non-ASD were obtained from Synapse (http://www.synapse.org) with permission under the accession number syn4587609. The RNA-seq and genotyping data of SCZ and non-SCZ were also obtained from Synapse with permission under the accession number syn4923029. The identified circQTLs/*trans*-eQTLs and the related data were deposited in Supplementary Data 1–8 or GitHub at https://github.com/TreesLab/circQTL_ASD. For the SCZ samples, the full list of the constructed circQTL-circRNA-*trans*-eGene axes (979,013 axes) based on all circQTLs was deposited at http://treeslab1.genomics.sinica.edu.tw/trans-eQTL_causality_SCZ/.

## Code availability

Code used for data analysis is publicly accessible at https://github.com/TreesLab/circQTL_ASD.
