# Supplementary material for: Trans-genetic effects of circular RNA expression quantitative trait loci and potential causal mechanisms in autism
Source: Mol Psychiatry. 2022 Aug 12;27(11):4695–706. doi: 10.1038/s41380-022-01714-4 (PMC9734057; doi:10.1038/s41380-022-01714-4)
Supplement: Supplementary file 1 — Supplementary Information [file 41380_2022_1714_MOESM1_ESM.docx]

**SUPPLEMENTARY INFORMATION**

***Trans*-genetic effects of circular RNA expression quantitative trait loci and potential causal mechanisms in autism**

**Te-Lun Mai^1,2^, Chia-Ying Chen^1^, Yu-Chen Chen^1^, Tai-Wei Chiang^1^, and** **Trees-Juen Chuang^1*^**

^1^Genomics Research Center, Academia Sinica, Taipei, Taiwan.

^2^Department of Life Science, National Taiwan University, Taipei, Taiwan

*Correspondence and requests for materials should be addressed to T.-J.C. (email: trees@gate.sinica.edu.tw).

These authors contributed equally: Te-Lun Mai, Chia-Ying Chen, Yu-Chen Chen.

| Supplementary Methods | Pages 2-10 |
| --- | --- |
| Supplementary Table 1 | Page 11 |
| Supplementary Figures 1-9 | Pages 12-23 |
| Supplementary Data legends | Page 24 |
| Supplementary References | Pages 25-27 |

**Supplementary Methods**

**Identification of circQTLs/*trans*-eQTLs**

The RNA-seq data of ASD and non-ASD brain samples [1] and the corresponding genotype data [2] were obtained from Synapse (http://www.synapse.org) with permission under the accession number syn4587609. The RNA-seq data were derived from 236 postmortem samples (85, 73, and 78 front cortex (FC), temporal cortex (TC), and cerebellar vermis (CV) samples). In our previous study [3], we had performed NCLscan [4], which was shown to exhibit the greatest precision among publicly-available tools [4-7], to identify circRNAs according to the same Synapse RNA-seq data used in this study and the Ensembl annotation (version 90) based on the human reference genome (GRCh38). For accuracy, samples were not considered if the numbers of circRNAs detected in the samples were below one standard deviation from the mean of the sample set. A total of 202 samples (73 FC, 61 TC, and 68 CV samples) and 53,427 previously-identified circRNAs [3] were utilized in this study. For minimizing potential spurious events, we only considered the 1,060 circRNAs that were detected in more than 50% of the samples examined [3]. This study considered RNA-seq data from cortex samples (73 FC and 61 TC samples) only because cortex was reported to be more selectively vulnerable to transcriptomic alternations than CV [1]. The genotype data were derived from 79 individuals, including 40 ASD patients and 39 non-ASD-affected controls [2]. Since eQTL analysis required both RNA-seq and genotype data from the same individuals, RNA-seq data from 105 samples were examined in the following analyses (Fig. 1b and Supplementary Data 1). The expression levels of circRNAs were measured by RPM [8] and then adjusted for the effects of covariates, including the corresponding host gene expression and biological/technical factors (brain region, diagnosis (ASD/non-ASD), age, sex, genetic ancestry (the first five principal components (PC1~PC5) based on the examined genotype data), RNA quality (RNA integrity number; RIN), post-mortem interval (PMI), sequencing batch, and brain bank, using a linear model. The expression levels of genes were normalized using the following three steps. First, we calculated the read counts of genes using the RSEM tool [9] based on the STAR [10] alignment results. Second, we calculated the log_2_ normalized FPKM (the fragments per kilobase million mapped reads) values, which accounted for gene read counts, GC content, gene length, and library size, using the cqn package in R software [11]. Third, the effects of the abovementioned biological/technical covariates were regressed out using a linear model. The covariate-adjusted expression levels of circRNAs/genes were deposited in Supplementary Data 1.

Imputation genotype data (39,166,259 SNPs) were downloaded using the Synapse command line client. For accuracy, we only considered the SNPs that simultaneously had imputed quality score (R^2^) ≥ 0.8, minor allele frequency (MAF) ≥ 0.01, and number of individuals with the minor allele (heterozygotes and minor allele homozygotes) ≥ 3. We thus retained 7,559,661 SNPs for the following eQTL analysis. We limited our analysis to SNPs in ± 200 kb nucleotides of each back-splice site (a total of 740,391 SNPs) and evaluated circQTLs by testing the correlations between the imputed genotype dosages and covariate-adjusted circRNA expression using Matrix eQTL with an additive linear model [12]. To correct the *P* value regardless of the distribution of circRNA expression and statistical test, a permutation test was conducted to estimate the empirical *P* value (emp*P*) for each uncorrected circQTL SNP by performing 10,000 permutations with randomly sampling labels of the circRNA expression matrix. The emp*P* value for each uncorrected circQTL *P* value (*P*_circQTL_) was calculated as emp*P* = $\frac{\text{1+}\sum_{i=1}^{\text{10,000}} \text{number of (}P_{i}<P_{circQTL}\text{)}}{\text{10,001}}$ . After that, to identify circQTL-containing circRNAs, we calculated *q* value for each circRNA using Storey’s method [13] based on the distribution of minimal emp*P* values of all circRNAs. A circRNA was defined as a circQTL-containing circRNA if the *q* value of the circRNA was less than 0.05. For each circQTL-containing circRNA, only the associated circQTLs with emp*P* < 0.005 were retained. A total of 4,729 circQTLs (associated 389 circRNAs) based on the 105 cortex (FC and TC) samples were identified (Fig. 1b; Supplementary Data 2). We further performed conditional and joint (COJO) analysis based on GCTA-COJO [14] to identify independent circQTLs within highly associated regions. We thus identified 605 independent circQTLs (associated with 389 circRNAs) based on the 105 cortex samples, respectively (Fig. 1b; Supplementary Data 2). Of the independent circQTLs, the most significant circQTL SNP for each circRNA was defined as the “max-circQTL”. For each circQTL-containing circRNA, the SNP with the minimal uncorrected *P* value larger than 0.05 was selected as a negative control (i.e., non-circQTL).

We examined whether a circQTLs was also a *trans*-eQTL affecting the expression of *trans*-eGenes using Matrix eQTL with an additive linear model. Such circQTL SNPs and *trans*-eGenes should be located on different chromosomes or the same chromosome separated by a distance greater than 5Mb (from the SNP site to the transcription start site of the *trans*-eGene). To correct the *P* value regardless of the distribution of remote gene expression and statistical test, a permutation test was conducted to estimate the empirical *P* value (emp*P*) for each uncorrected circQTL SNP by performing 10,000 permutations with randomly sampling labels of the expression matrix of remote genes. The emp*P* value for each uncorrected *trans*-eQTL *P* value (*P_trans_*_-eQTL_) was calculated as emp*P* = $\frac{1+\sum_{i=1}^{10,000} \mathrm{number} \mathrm{of} (P_{i}<P_{\text{trans}\text{-eQTL}})}{10,001}$. The *trans*-effect of a circQTL on the expression of a *trans*-eGene (or a circQTL-*trans*-eGene pair) was determined if *P_trans_*_-eQTL_ was less than 10^-4^ and emp*P* was less than 0.005 simultaneously. The *trans*-effects of non-circQTLs on the expression of remote genes were also examined using the same procedures mentioned above.

**Detection of reverse complementary sequences (RCSs)**

The criteria for detecting RCSs were similar to those presented in a previous study [15]. For a circRNA, both flanking sequences (± 200 kb nucleotides of the back-splice site) were aligned each other using BLAST[16] with parameters –task blastn –word_size 11 –strand minus. The potential RCSs should be simultaneously satisfied the following rules: bitscore> 100, alignment length>50 bp, and identity>80%.

**Mediation and causal testing**

We conducted mediations analysis using the mediation package [17] downloaded from the Comprehensive R Archive Network (CRAN) at https://cran.r-project.org/web/packages/mediation/index.html. To test the mediation effects of *trans*-eQTLs, the mediation package was performed with circQTL (*trans*-eQTL) as the “exposure,” the normalized expression levels of circRNAs as the “mediator,” and the normalized expression levels of *trans*-eGenes as the “outcome”. The proportion of mediation for the circQTL was the proportion of causal mediation effect in the total effect (i.e., causal mediation effect + direct effect) [17]. For the partial correlation analysis, we calculated Spearman correlation coefficient between the normalized expression levels of circRNAs and *trans*-eGenes with controlling for the circQTL (*trans*-eQTL) SNPs. The residuals after adjusting for the circQTLs would be significantly correlated (post-adjustment *P*<0.05), if there was a causal relationship between circRNA expression and *trans*-eGene expression (i.e., the axes passing the partial correlation test). The partial correlation analysis was performed using the ppcor program in R [18]. In Fig. 2f, we evaluated the significant difference between two independent Spearman’s correlations using a two-tailed Z-score test with the paired.r function in the *psych* R library.

The CIT package [19] was downloaded from CRAN at https://cran.r-project.org/web/packages/cit/index.html. A CIT-passing circQTL-*trans*-eGene pair (CIT *P*<0.05 and FDR (*q*) <0.05) (Supplementary Data 3 for the ASD samples and Supplementary Data 5 for the SCZ samples) represents that the circQTL has a causal effect on the diagnosis status (ASD vs. non-ASD or SCZ vs. non-SCZ) through the *trans*-eGene expression, which should simultaneously satisfy the following conditions: (1) the circQTL is associated with the diagnosis status, (2) the circQTL is associated with the *trans*-eGene mediator after adjusting for the diagnosis status, (3) the *trans*-eGene mediator is associated with the diagnosis status after adjusting for the circQTL, and (4) the circQTL is independent of the diagnosis status after adjusting for the *trans*-eGene mediator. Of note, the expression levels of genes were measured by the similar processes sated above with regressing out the effects of all biological/technical covariates except for diagnosis. The *P* value of CIT is defined as the maximum of the above four-component test *P* values. The FDR (*q*) values were further estimated using CIT-provided permutation test with default parameters. Illustration of networks was plotted by the Cytoscape package (https://cytoscape.org/).

To test whether the circRNA-*trans*-eGene associations were mediated by the expression of miRNAs, we first extracted miRNA expression data [20] based on the 63 cortex samples (30 ASD and 33 non-ASD samples) overlapped with the samples examined in our eQTL analysis. The miRNA expression was normalized using the following three steps. First, the miRNA read counts were obtained from Wu et al.’s study [20] upon request. Second, the log_2_ normalized FPKM values were calculated by the cqn package with accounting for miRNA read counts, GC content, miRNA length, and library size. Third, the effects of the biological/technical covariates (including diagnosis, brain region, age, sex, genetic ancestry (PC1~PC5), RIN, PMI, SeqBatch, BrainBank, and PropReadOnExon) were regressed out using a linear model. We then calculated Spearman correlation coefficient between the circRNA expression and the *trans*-eGene expression and retained the circRNA-miRNA-*trans*-eGene axes with significant circRNA-*trans*-eGene correlations (Spearman’s *P*<0.05). We reevaluated the correlations by regressing out the miRNA expression using a partial correlation analysis. If the miRNAs acted as a mediator for the circRNA-*trans*-eGene associations, the residuals after adjusting for the miRNA expression would be unrelated (post-adjustment *P*>0.05). The partial correlation analysis was performed using the ppcor package in R [18].

**Replication analysis of circQTLs/*trans*-eQTLs based on the CMC database**

We first obtained the circRNA junction counts from Liu et al. [21] upon request, in which the circRNAs were identified according to RNA-seq data from the postmortem brain samples of dorsolateral prefrontal cortex (DLPFC) from the CommonMind Consortium (CMC) database [22] (Synapse ID: syn4923029). Here a total of 414 genetically inferred Caucasians [22] (209 SCZ and 205 control samples) were considered in this study (see also Supplementary Fig. 1). To increase the stringency of sample consistency, a sample was excluded if the number of the identified circRNAs of this sample was one standard deviation below the mean of the sample set. After that, 349 samples (171 SCZ patients and 178 controls; Supplementary Data 4) including 1,020,387 circRNAs were retained. To minimize potentially spurious events, 18,322 circRNAs detected in more than half of the 349 samples were considered. For accuracy, we only considered the 15,666 circRNAs that agreed to well-annotated exon boundaries of co-linear transcripts (Ensembl annotation; version 90). The corresponding genotype data and the related biological/technical factors were downloaded from the CMC Knowledge Portal [22] at https://www/synapse.org/#!Synapse:syn3275221. The gene read counts were downloaded from the CMC database (Synapse ID: syn3346749). Like the processes mentioned in the analysis of the ASD samples, the expression levels of circRNAs were calculated using RPM. The effects of covariates including the corresponding host gene expression and the CMC-provided biological/technical factors (diagnosis (SCZ/non-SCZ), age, sex, RIN, PMI, sequencing batch, and institution) were regressed out. The expression levels of genes were measured and normalized by the same steps stated above. Regarding the 349 samples, a total of 7,386,012 SNPs that simultaneously satisfied imputed quality score (R^2^) ≥ 0.8 and MAF ≥ 0.01 were considered for the eQTL analysis. Using the same procedures used for identifying circQTLs/*trans*-eQTLs in the ASD samples, we finally identified 333,548 circQTLs (18,338 independent circQTLs) associated with 9,622 circRNAs, which constructed 476,812 circQTL-circRNA associations (19,201 circQTL-circRNA associations based on the independent circQTLs) (Supplementary Fig. 1 and Supplementary Data 4). Of the 333,548 circQTLs, 165,626 circQTLs were also *trans*-eQTLs associated with 979,013 circQTL-circRNA-*trans*-eGene axes; of the 18,338 independent circQTLs, 9,975 circQTLs were also *trans*-eQTLs associated with 64,685 circQTL-circRNA-*trans*-eGene axes (Supplementary Fig. 2f and Supplementary Data 5).

We observed that 19,879 out of the 64,685 (30.7%) axes passed the mediation test (Supplementary Fig. 2b and Supplementary Data 5). The majority (66.7%, 13,256 axes) of the 19,879 axes exhibited that at least 10% of the circQTL-*trans*-eGene associations were mediated by the expression of circRNAs; in some cases (518 axes), the proportion of mediation of the circQTL-*trans*-eGene association by the circRNAs was even more than 30% (Supplementary Fig. 2b). Like the trends observed in the analyses for the ASD samples (Fig. 2), the mediation effects of the circQTLs (*trans*-eQTLs) on circRNA expression were positively correlated with the magnitude of circRNA-*trans*-eGene correlation of expression profile, regardless of whether the circQTL SNPs were controlled (Supplementary Figs. 2c and 2d). The *P*_ACME_ values were more strongly correlated with the post-adjustment *P* values than with the correlation *P* values before SNP adjustment (Supplementary Figs. 2e). These results also supported the mediation effects of *trans*-eQTLs for the SCZ samples. Considering all circQTLs (333,548 circQTLs; Supplementary Fig. 1), 165,626 circQTLs were also *trans*-eQTLs associated with 979,013 circQTL-circRNA-*trans*-eGene axes; of the 979,013 axes, 271,298 passed the mediation test and 215,866 passed the MPT (Supplementary Fig. 2f).

On the basis of the 349 samples, we used the nlme package in R software (version 3.4.2) to performed a linear mixed effects (LME) model and then identified differentially expressed (DE)-circRNAs between SCZ and non-SCZ samples with controlling for biological/technical factors including age, sex, RIN, PMI, host gene expression, sequencing batch, and institution as lme(RPM~ diagnosis + age + sex + RIN + PMI + host gene expression + sequencing.batch + institution, rand = ~1|individual ID). A total of 203 circRNAs with *P*<0.01 were identified as DE-circRNAs (Supplementary Data 6).

**TWAS analyses**

ASD GWAS [23] (18,381 cases and 27,969 controls) and SCZ GWAS [24] (67,390 cases and 94,015 controls) SNPs were downloaded from https://doi.org/10.6084/m9.figshare.14671989.v1 and https://doi.org/10.6084/m9.figshare.14672178.v1, respectively. Considering the Synapse and CMC datasets of SNP-expression weights, the TWAS analyses were performed using the FUSION package (http://gusevlab.org/projects/fusion/) [25]. *P* values of association statistics were calculated using Bonferroni correction.

**Identification of disease-associated SNP clumps**

On the basis of the GWAS data for ASD and SCZ, linkage disequilibrium (LD)-based SNP clumping was performed by PLINK (version 1.07) [26] with the following parameters: -clump-p1 0.001 -clump-p2 0.05 -clump-r2 0.5 -clump-kb 250. LD information was downloaded from the 1000 Genomes Project (phase 3) at http://ftp.ensembl.org/pub/data_files/homo_sapiens/GRCh38/variation_genotype/. A circRNA event overlapped with a clump if one of the back-splice sites (donor or acceptor sites) were located within the clump. The identified ASD- and SCZ-associated SNP clumps were deposited in Supplementary Data 2 and 4, respectively.

**Enrichment analyses of the CIT-passing *trans*-eGenes**

The SFARI [27] gene list was downloaded from https://gene.sfari.org/ (SFARI 08-07-2020 release). The AutismKB gene list (a core dataset with 228 high-confidence ASD-associated genes) was downloaded from the AutismKB 2.0 database [28]. The gene set of the high-confidence ASD genetic risk genes (102 genes) was downloaded from the Satterstrom *et al*.’s study [29]. The lists of genes encoding PSD proteins and targets of FMRP, RBFOX1, and ELAVL1 were downloaded from Lee et al.’s study [2]. The list of DEGs in ASD was downloaded from Parikshak et al.’s study [1]. The probability of ASD risk and pLI score for each gene were downloaded from http://asd.princeton.edu/ and https://gnomad.broadinstitute.org/ (loss-of-function curation results; v2.1.1), respectively. The list of DEGs in a cell type-specific manner was downloaded from Velmeshev et al.’s study [30]. The list of genes previously implicated in SCZ was downloaded from the SzGene database [31]. The list of DEGs in SCZ was downloaded from Fromer et al.’s study [22]. For gene set enrichment analyses, we used 20,070 protein-coding genes as the background set. The 20,070 genes were selected because they were expressed in the cortex samples based on the RNA-seq data examined in this study. We took the analysis of SFARI gene enrichment for the CIT-passing *trans*-eGenes as an example. We created a two-way contingency table with rows containing numbers of SFARI and non-SFARI genes and columns containing numbers of CIT-passing *trans*-eGenes and genes that were not CIT-passing *trans*-eGenes. The statistical significance and odds ratio were determined using one-tailed Fisher’s exact test with the *fisher.test* R function. GO analysis was performed using the ToppFun module of ToppGene Suite software [32] at https://toppgene.cchmc.org/enrichment.jsp (version 2020-Sep-08 01:39).

**Determination of miRNA binding sites**

CLIP-supported Ago binding sites on hg19 assembly were downloaded from ENCORI[33] at http://starbase.sysu.edu.cn/. The liftOver tool [34] was employed to obtain the genomic coordinates of binding sites on the GRCh38 assembly. The miRNA-circRNA and miRNA-mRNA interactions supported by CLIP-seq data were also extracted from ENCORI. These data were retrieved through ENCORI-provided Web APIs. Of note, a considered miRNA binding site should be supported by at least one Ago CLIP-seq experiments. CircRNA-miRNA-mRNA interactions were determined according to the common miRNA target sites of the circRNAs and mRNAs. The in-house software (CircMiMi) for constructing circRNA-miRNA-mRNA interactions is available at https://github.com/TreesLab/CircMiMi. In addition, the experimentally-supported miRNA-mRNA axes were downloaded from miRTarBase (version 7.0) [35] and DIANA-TarBase (version 8) [36].

**Validation of the correlations between the expression of circHOMER1a, miR-641, and *MBNL3***

Normal human astrocyte (NHA) cell line was purchased from Gibco (cat. #N7805100) and cultured in human astrocytes growth medium supplemented with 10% fetal calf serum and 1% penicillin streptomycin (Gibco, cat. #15140-122) at 37^o^C and placed in a humidified atmosphere containing 5% CO_2_.

To overexpress circHOMER1a, the circularized sequences (Exons 2-5; see also Supplementary Fig. 7a) were amplified in NHA cells and the PCR product were inserted into homemade pCMV-circ plasmids, which were constructed with the repetitive elements in the designated orientations as previously described ^1^. The construct was validated by Sanger sequencing. After cells were seeded in 6-well plates (2.5 x 10^5^ cells/well) for 24 hours, cells were transfected with Dsi-RNA (10 nM), miRNA mimics/inhibitor (50 nM), and plasmids (500 ng/well) using TransIT-X2 dynamic delivery system (Mirus, cat. #MIR 6003), respectively.

For qRT-PCR analyses, total RNA and miRNA were extracted from NHA cells using TRIzol Reagent (Life technologies, cat. #15596018) and the PureLink RNA Mini Kit (Thermo Fisher Scientific, cat. #12183018A). Total RNA and miRNA were then reverse transcribed with SuperScript IV Reverse Transcriptase kit (Thermo Fisher Scientific, cat. #18090010) and miRCURY LNA RT kit (QIAGEN, cat. #339340), respectively. qRT-PCR was conducted on a QuantStudio 5 Real-Time PCR System (Applied Biosystems) to measure gene (or circRNA) expression and miRNA expression using Luminaris Color HiGreen qPCR Master Mix (Thermo Fisher Scientific, cat. #K0391) and miRCURY LNA SYBR Green PCR kit (QIAGEN, cat. #339346), respectively. *GAPDH* was used as the endogenous control for quantification of circRNA and mRNA. miRNA expression was normalized to *U6*. The relative expression levels of circRNA, miRNA, and mRNA were calculated by the comparative threshold cycle (2^-ΔΔCT^) method.

**Statistics**

For *cis*-/*trans*-eQTL analyses using Matrix eQTL package (Figs. 1c, 2a, 2g; Supplementary Figs. 1, 2a, 2f; Supplementary Data 2-5), normality was not formally tested for each circRNA/mRNA. We conducted a permutation test to correct the *P* values regardless of the distribution of circRNA expression and statistical test. For causality analyses using mediation test (Figs. 2b, 2g, 4a, 5a, 5b; Supplementary Figs. 2b, 2f, 6c; and Supplementary Data 3, 5) and CIT (Figs. 3b, 4a, 5b; Supplementary Fig. 6c; Supplementary Data 3, 5) (both are linear models), normality was not formally tested for each circRNA/mRNA. For accuracy, we also conducted a partial correlation analysis based on a nonparametric statistic (Spearman’s correlation analysis), which does not rest upon an assumption of normality, to examine the scenarios of a causal relationship between circRNA expression and *trans*-eGene expression (Figs. 4a, 5a, 5b, and Supplementary Fig. 6c) and a mediator of miRNAs for the circRNA-*trans*-eGene associations (Figs. 5a and 5b). The correlations between the mediation effects of the circQTLs on circRNA expression and the magnitude of circRNA-*trans*-eGene correlation of expression profile were also tested by Spearman’s correlation analysis (Figs. 2c, 2e, and Supplementary Figs. 2c and 2d). Significant difference between two independent correlations was evaluated using two-tailed Z-score test (Fig. 2f and Supplementary Fig. 2e). For Figures 1d, 3b-3d, and Supplementary Figure 6b, *P* values were determined using two-tailed Fisher’s exact test. *P* values were FDR adjusted across nine target groups for each gene list using Benjamini-Hochberg correction (Fig. 3b). For the expression comparison in experimental validation (Fig. 6), two-tailed *t*-tests were used. Equal variances were not formally tested. These tests were performed with the assumption of unequal variance.

**Sample size**

No statistical methods were used to pre-determine sample sizes to ensure adequate power, but the data used were downloaded from the newly released Synapse database [1, 2], which comprises a large human brain sample size of both genotype data and RNA-seq data (rRNA-depleted RNAs from total RNAs without poly(A)-selection) from ASD cases and controls. This is by far the largest sample size for circRNA studies in ASD. The genotype data and RNA-Seq data of ASD and control brains are public data sets. Sample sizes were predetermined in the previous studies [1, 2], which were sufficient for the molecular analyses.

**Supplementary Table 1.** The primer, Dsi-RNA, miRNA mimic, miRNA inhibitor sequences used in this study.

| **Name** | **Sequence** | **Note (company)** |
| --- | --- | --- |
| Dsi-circ-Homer1a_Sense | rArUrUrUrUrCrArCrArUrArGrGrGrArArCrArArCrCrUrATC | siRNA knockdown (IDT) |
| Dsi-circ-Homer1a_Antisense | rGrArUrArGrGrUrUrGrUrUrCrCrCrUrArUrGrUrGrArArArArUrGrG |  |
| miR-641_mimics_Sense | AAAGACAUAGGAUAGAGUCACCUC | miRNA mimics (BIOTOOLS) |
| miR-641_mimics_Antisense | GGUGACUCUAUCCUAUGUCUUUUU |  |
| miR-mimics-NC_Sense | UUCUCCGAACGUGUCAUGUTT |  |
| miR-mimics-NC_Antiense | ACGUGACACGUUCGGAGAATT |  |
| miR-641_inhibitor | GAGGUGACUCUAUCCUAUGUCUUU | miRNA inhibitor (BIOTOOLS) |
| miR-inhibitor-NC | CAGUACUUUUGUGUAGUACAA |  |
| *MBNL3*_F | CCTATGACTCCATCAATTCCAGC | qRT-PCR (IDT) |
| *MBNL3*_R | CGCATCAGTTTTGGGCCAAC |  |
| circHomer1a_F | GCCAAGGGCTGAACCAACTCAG |  |
| circHomer1a_R | GCATGCTTGCTGGTGGGTACCC |  |
| GAPDH_F | GGAGCGAGATCCCTCCAAAAT |  |
| GAPDH_R | GGCTGTTGTCATACTTCTCATGG |  |
| has-miR-641 | Cat no: YP00204411 | miRCURY LNA^TM^ miRNA PCR Assay (QIAGEN) |

**Supplementary Figure 1.** The pre-processes for the RNA-seq data, genotype data, and the corresponding circRNAs in the replication analysis of the SCZ brain samples (SCZ patients and controls) from the CMC database.

**Supplementary Figure 2.** Mediation analysis for the identified circQTL-circRNA-*trans*-eGene axes based on the independent circQTLs for the replication analysis in SCZ. **a** Schematic diagram representing direct (blue bold line with arrow) and mediation (red bold line with arrow) effects of circQTLs (*trans*-eQTLs) on the expression of *trans*-eGenes. **b** The significance levels (top) and effect sizes (beta values; bottom) of the direct (y axis) and mediation (x axis) effects for the 64,685 circQTL-circRNA-*trans*-eGene axes. *P*_ADE_ and *P*_ACME_ values represented the significance levels of the average direct (ADE) and average causal mediation (ACME) effects, which were determined by the mediation package. The dashed line represented *P* = 0.05 for mediation effects. Of the 64,685 circQTL-circRNA-*trans*-eGene axes, 19,879 axes (30.7%) passed the mediation test. For the top right panel, the numbers of axes were shown in parentheses. **c,d** The correlations between the percentages of circQTL-circRNA-*trans*-eGene axes passing the mediation test and the significance levels of the Spearman’s correlation of expression profile between circRNAs and *trans*-eGenes before **(c)** and after **(d)** SNP adjustment. **e** Correlations between mediation effects and the significance levels of circRNAs-*trans*-eGenes correlations before or after SNP adjustment. Statistical significance of Spearman’s rank correlation coefficient was denoted by black words. Significant difference between two independent correlations was evaluated using two-tailed Z-score test and denoted by red words. **f** A summary table of the identified circQTL-circRNA-*trans*-eGene axes passing mediation test or MPT.


**c**

**Supplementary Figure 3.** Associations between the internal modules of the identified circQTL-circRNA pairs and disease risk loci. **a,b** The quantile-quantile plots of the identified circQTL SNPs (left), the bar charts of the circQTLs overlapping with disease-related GWAS sites (with GWAS *P*<0.05, *P*<5×10^-5^, and *P*<5×10^-8^) (middle), and the bar charts of the circQTLs/circRNAs overlapping with disease-associated SNP clumps (right) for the analyses of the (**a**) ASD and (**b**) SCZ samples. The ASD- or SCZ-associated SNP clumps were calculated using PLINK with the parameter p1 values of 0.001, 5×10^-5^, and *P*<5×10^-8^ (see Supplementary Methods). **c** Results from the TWAS-prioritized circRNAs in the SCZ samples using TWAS-FUSION. DE-circRNAs in SCZ were shown in arrows. The sign of TWAS-FUSION z-scores represented the direction of effect.

**Supplementary Figure 4.** The associations of the internal modules of the identified **(a)** circQTL-circRNA axes and **(b)** circQTL-circRNA-*trans*-eGene axes with ASD/SCZ. For **(a)**, the numbers of the ASD- and SCZ-associated internal modules (i.e., circQTLs or circRNAs) were denoted in parentheses. For **(b)**, the numbers of the ASD- and SCZ-associated internal modules (i.e., circQTLs, circRNAs, or *trans*-eGenes) were denoted in parentheses, in which the numbers of internal modules involved in all axes and the axes passing MPT were denoted in green and orange, respectively.

**Supplementary Figure 5.** Gene Ontology (GO) analysis of the CIT-passing genes in terms of **(a)** molecular function, **(b)** biological process, and **(c)** cellular component. The dashed lines represented Bonferroni-corrected *P*=0.05.

**Supplementary Figure 6.** Inference of causal relations between circQTLs (*trans*-eQTLs), *trans*-eGenes, and ASD diagnosis by CIT for the replication analysis in SCZ. **a** The circQTL-*trans*-eGene pairs (476 pairs) passing CIT with the propagation path from circQTL SNPs (398 circQTLs) to ASD diagnosis via *trans*-eGene (97 genes) expression. **b** Enrichment analysis of SCZ-relevant genes for the CIT-passing genes. SCZ-relevant genes included ASD risk genes from SzGene and DEGs in SCZ. *P* values were determined using one-tailed Fisher’s exact test. The dashed lines represented *P=*0.05. The enrichment odd ratios with *P*≤0.05 were shown in parentheses. **c** Identification of potential circQTL-circRNA-*trans*-eGene-ASD diagnosis propagation paths (right) by integrating CIT-passing circQTL-*trans*-eGene pairs (left) with MPT-passing circQTL-circRNA-*trans*-eGene axes (middle). **d** The 263 identified circQTL-circRNA-*trans*-eGene-ASD diagnosis propagation networks plotted by the Cytoscape package. The SCZ-relevant *trans*-eGenes were shown. The black line with arrows represented that the circQTLs SNPs were the significantly SCZ-related GWAS sites.

**Supplementary Figure 7.** Numbers of the circQTL-circRNA-*trans*-eGene axes passing mediation test and the partial correlation test for the **(a)** ASD samples and **(b)** SCZ samples.

**Supplementary Figure 8.** Effect sizes (odds ratios) of ASD-related GWAS SNPs [23] plotted against their corresponding *trans*-effects (beta) on *trans*-eGenes. For each ASD-related circQTL, the most significant effect of the circQTL on the *trans*-eGene is selected (Supplementary Data 3). Error bars represent the standard errors of SNP effects. The right panel represents an example of the circQTL (rs143905310) in a SFARI gene (*AGTR2*). Center values and error bars represent mean values and s.e.m., respectively.

**Supplementary Figure 9.** Expression variance (*r^2^*) explained by a circQTL for each *trans*-eGene that is also a DEG in ASD. The maximum variance explained by a circQTL for each DEG is selected. The DEGs and the corresponding log_2_(fold change) values are downloaded from [1]. The red dots represent the SFARI genes. The right panel represents an example of the circQTL (rs6741911) in the DEG with a highest fold change (*HSPA6*). Center values and error bars represent mean values and s.e.m., respectively.

**Supplementary Data Legends**

**Supplementary Data 1.** The ASD brain samples used in this study and the circRNA expression profiles for the used samples.

**Supplementary Data 2**. The identified circQTL-circRNA associations based on the 105 samples from two cortical regions (FC and TC) (related to Fig. 1).

**Supplementary Data 3.** The identified circQTLs that were also *trans*-eQTLs and the causality analyses of the constructed circQTL-circRNA-*trans*-eGene axes in ASD (related to Figs. 2, 4, 5 and Supplementary Figs. 8, 9).

**Supplementary Data 4.** The SCZ brain samples used in this study and the identified circQTL-circRNA associations (related to Supplementary Fig. 1).

**Supplementary Data 5.** The identified circQTLs that were also *trans*-eQTLs and the causality analyses of the constructed circQTL-circRNA-*trans*-eGene axes in SCZ (related to Supplementary Figs. 2 and 5).

**Supplementary Data 6**. Differentially expressed circRNAs (DE-circRNAs) in ASD and SCZ.

**Supplementary Data 7.** TWAS-prioritized circRNAs in the SCZ samples.

**Supplementary Data 8.** The identified circQTL-circRNA-miRNA-*trans*-eGene regulatory axes (related to Fig. 5).

**Supplementary References**

1. Parikshak NN, Swarup V, Belgard TG, Irimia M, Ramaswami G, Gandal MJ *et al.* Genome-wide changes in lncRNA, splicing, and regional gene expression patterns in autism. *Nature* 2016; **540**(7633)**:** 423-427.

2. Lee C, Kang EY, Gandal MJ, Eskin E, Geschwind DH. Profiling allele-specific gene expression in brains from individuals with autism spectrum disorder reveals preferential minor allele usage. *Nat Neurosci* 2019; **22**(9)**:** 1521-1532.

3. Chen YJ, Chen CY, Mai TL, Chuang CF, Chen YC, Gupta SK *et al.* Genome-wide, integrative analysis of circular RNA dysregulation and the corresponding circular RNA-microRNA-mRNA regulatory axes in autism. *Genome research* 2020; **30**(3)**:** 375-391.

4. Chuang TJ, Wu CS, Chen CY, Hung LY, Chiang TW, Yang MY. NCLscan: accurate identification of non-co-linear transcripts (fusion, trans-splicing and circular RNA) with a good balance between sensitivity and precision. *Nucleic acids research* 2016; **44**(3)**:** e29.

5. Zeng X, Lin W, Guo M, Zou Q. A comprehensive overview and evaluation of circular RNA detection tools. *PLoS computational biology* 2017; **13**(6)**:** e1005420.

6. Chen CY, Chuang TJ. Comment on "A comprehensive overview and evaluation of circular RNA detection tools". *PLoS computational biology* 2019; **15**(5)**:** e1006158.

7. Chen CY, Chuang TJ. NCLcomparator: systematically post-screening non-co-linear transcripts (circular, trans-spliced, or fusion RNAs) identified from various detectors. *BMC bioinformatics* 2019; **20**(1)**:** 3.

8. Veno MT, Hansen TB, Veno ST, Clausen BH, Grebing M, Finsen B *et al.* Spatio-temporal regulation of circular RNA expression during porcine embryonic brain development. *Genome biology* 2015; **16:** 245.

9. Li B, Dewey CN. RSEM: accurate transcript quantification from RNA-Seq data with or without a reference genome. *BMC bioinformatics* 2011; **12:** 323.

10. Dobin A, Davis CA, Schlesinger F, Drenkow J, Zaleski C, Jha S *et al.* STAR: ultrafast universal RNA-seq aligner. *Bioinformatics* 2013; **29**(1)**:** 15-21.

11. Hansen KD, Irizarry RA, Wu Z. Removing technical variability in RNA-seq data using conditional quantile normalization. *Biostatistics* 2012; **13**(2)**:** 204-216.

12. Shabalin AA. Matrix eQTL: ultra fast eQTL analysis via large matrix operations. *Bioinformatics* 2012; **28**(10)**:** 1353-1358.

13. Storey JD, Tibshirani R. Statistical significance for genomewide studies. *Proceedings of the National Academy of Sciences of the United States of America* 2003; **100**(16)**:** 9440-9445.

14. Yang J, Ferreira T, Morris AP, Medland SE, Genetic Investigation of ATC, Replication DIG *et al.* Conditional and joint multiple-SNP analysis of GWAS summary statistics identifies additional variants influencing complex traits. *Nature genetics* 2012; **44**(4)**:** 369-375, S361-363.

15. Ivanov A, Memczak S, Wyler E, Torti F, Porath HT, Orejuela MR *et al.* Analysis of intron sequences reveals hallmarks of circular RNA biogenesis in animals. *Cell reports* 2015; **10**(2)**:** 170-177.

16. Altschul SF, Gish W, Miller W, Myers EW, Lipman DJ. Basic local alignment search tool. *Journal of molecular biology* 1990; **215**(3)**:** 403-410.

17. Tingley D YT, Hirose K, Keele L, Imai K. mediation: R Package for Causal Mediation Analysis. *Journal of Statistical Software* 2014; **59**(5)**:** 1-38.

18. Kim S. ppcor: An R Package for a Fast Calculation to Semi-partial Correlation Coefficients. *Commun Stat Appl Methods* 2015; **22**(6)**:** 665-674.

19. Millstein J, Chen GK, Breton CV. cit: hypothesis testing software for mediation analysis in genomic applications. *Bioinformatics* 2016; **32**(15)**:** 2364-2365.

20. Wu YE, Parikshak NN, Belgard TG, Geschwind DH. Genome-wide, integrative analysis implicates microRNA dysregulation in autism spectrum disorder. *Nat Neurosci* 2016; **19**(11)**:** 1463-1476.

21. Liu Z, Ran Y, Tao C, Li S, Chen J, Yang E. Detection of circular RNA expression and related quantitative trait loci in the human dorsolateral prefrontal cortex. *Genome biology* 2019; **20**(1)**:** 99.

22. Fromer M, Roussos P, Sieberts SK, Johnson JS, Kavanagh DH, Perumal TM *et al.* Gene expression elucidates functional impact of polygenic risk for schizophrenia. *Nat Neurosci* 2016; **19**(11)**:** 1442-1453.

23. Grove J, Ripke S, Als TD, Mattheisen M, Walters RK, Won H *et al.* Identification of common genetic risk variants for autism spectrum disorder. *Nature genetics* 2019; **51**(3)**:** 431-444.

24. Ripke S WJ, O’Donovan MC. Mapping genomic loci prioritises genes and implicates synaptic biology in schizophrenia. *medRxiv* 2021**:** 2020:2020.2009.2012. 20192922.

25. Gusev A, Ko A, Shi H, Bhatia G, Chung W, Penninx BW *et al.* Integrative approaches for large-scale transcriptome-wide association studies. *Nature genetics* 2016; **48**(3)**:** 245-252.

26. Purcell S, Neale B, Todd-Brown K, Thomas L, Ferreira MA, Bender D *et al.* PLINK: a tool set for whole-genome association and population-based linkage analyses. *American journal of human genetics* 2007; **81**(3)**:** 559-575.

27. Abrahams BS, Arking DE, Campbell DB, Mefford HC, Morrow EM, Weiss LA *et al.* SFARI Gene 2.0: a community-driven knowledgebase for the autism spectrum disorders (ASDs). *Mol Autism* 2013; **4**(1)**:** 36.

28. Yang C, Li J, Wu Q, Yang X, Huang AY, Zhang J *et al.* AutismKB 2.0: a knowledgebase for the genetic evidence of autism spectrum disorder. *Database : the journal of biological databases and curation* 2018; **2018**.

29. Satterstrom FK, Kosmicki JA, Wang J, Breen MS, De Rubeis S, An JY *et al.* Large-Scale Exome Sequencing Study Implicates Both Developmental and Functional Changes in the Neurobiology of Autism. *Cell* 2020; **180**(3)**:** 568-584 e523.

30. Liu X, Finucane HK, Gusev A, Bhatia G, Gazal S, O'Connor L *et al.* Functional Architectures of Local and Distal Regulation of Gene Expression in Multiple Human Tissues. *American journal of human genetics* 2017; **100**(4)**:** 605-616.

31. Allen NC, Bagade S, McQueen MB, Ioannidis JP, Kavvoura FK, Khoury MJ *et al.* Systematic meta-analyses and field synopsis of genetic association studies in schizophrenia: the SzGene database. *Nature genetics* 2008; **40**(7)**:** 827-834.

32. Chen J, Bardes EE, Aronow BJ, Jegga AG. ToppGene Suite for gene list enrichment analysis and candidate gene prioritization. *Nucleic acids research* 2009; **37**(Web Server issue)**:** W305-311.

33. Li JH, Liu S, Zhou H, Qu LH, Yang JH. starBase v2.0: decoding miRNA-ceRNA, miRNA-ncRNA and protein-RNA interaction networks from large-scale CLIP-Seq data. *Nucleic acids research* 2014; **42**(Database issue)**:** D92-97.

34. Hinrichs AS, Karolchik D, Baertsch R, Barber GP, Bejerano G, Clawson H *et al.* The UCSC Genome Browser Database: update 2006. *Nucleic Acids Res* 2006; **34**(Database issue)**:** D590-598.

35. Huang HY, Lin YC, Li J, Huang KY, Shrestha S, Hong HC *et al.* miRTarBase 2020: updates to the experimentally validated microRNA-target interaction database. *Nucleic acids research* 2020; **48**(D1)**:** D148-D154.

36. Karagkouni D, Paraskevopoulou MD, Chatzopoulos S, Vlachos IS, Tastsoglou S, Kanellos I *et al.* DIANA-TarBase v8: a decade-long collection of experimentally supported miRNA-gene interactions. *Nucleic acids research* 2018; **46**(D1)**:** D239-D245.
